# Pathogenicity and virulence of *Xanthomonas* in plants: Toward understanding specificity and achieving durable genetic resistance

**DOI:** 10.1080/21505594.2026.2758037

**Published:** 2026-11-05

**Authors:** Yumino Sasaki, Palash Ghosh, Neha Potnis, Adam J. Bogdanove

**Affiliations:** a Plant Pathology and Plant-Microbe Biology Section, School of Integrative Plant Science, Cornell University, Ithaca, NY, USA; b Department of Entomology and Plant Pathology, Auburn University, Auburn, AL, USA

**Keywords:** Host-pathogen interactions, virulence factors, host-specificity, tissue-specificity, susceptibility genes, plant immunity, engineered resistance

## Abstract

Plant pathogens in the bacterial genus *Xanthomonas* infect over 400 host species, including many economically important crop plants. In this review, we provide an overview of the genus, then highlight the infection and virulence strategies of *Xanthomonas* pathogens: how they survive in the phyllosphere, enter plant tissue, manipulate host immunity and physiology, and regulate virulence pathways, with a particular focus on host and tissue specificity. We then focus on host immunity against *Xanthomonas* pathogens, including cell surface, intracellular, and *Xanthomonas*-specific immunity, addressing the potential of discoveries in each compartment as foundations for engineered defense. While significant progress has been made, continued research on the diversity of *Xanthomonas* virulence mechanisms and counteracting plant defense will be instrumental for such engineering, promising at the same time deeper insight into pathogen-host interactions.

*Xanthomonas* is a large genus of plant-associated, primarily plant-pathogenic bacteria. Individually, members of the genus exhibit host species and often host genotype specificity. They also exhibit tissue specificity, with some invading xylem (“vascular” pathogens) and others colonizing mesophyll or fruit epidermal tissue (“non-vascular”). Within a *Xanthomonas* species, strains differing in host or tissue specificity are distinguished using the infra-subspecific designation “pathovar” (pv.). Collectively, different species and pathovars within the genus infect at least 400 plant species. These include many important crop species, leading to substantial economic damage and threatening food security worldwide, particularly for subsistence farmers in the global South [1]. An example is Xanthomonas wilt of banana in Sub-Saharan Africa, where for many people banana is a staple. Recent modeling predicts a production decrease of 55% or more within 10 years if control is not achieved, resulting in an estimated 25 billion dollars of loss and, among the populations for whom banana is a major source of calories, a nearly 5% increase in the number of people at risk of hunger [2]. *Xanthomonas* is also responsible for several diseases of ornamental crops, adding to the negative economic and environmental impact of the genus. An example is bacterial blight of geranium, which causes 10–15% loss across the industry and often 100% loss in individual greenhouse operations [3].

As is true of other bacterial pathogens of plants, chemical, cultural, and biological controls for *Xanthomonas* are limited, and host genetic resistance faces challenges. Qualitative resistance conferred by single dominant genes tends to be short-lived as pathogen populations evolve to evade recognition or overcome plant defense. Quantitative resistance conferred by multiple unlinked genes is typically more stable, but often intractable in breeding. These constraints have driven a focus on the genetic and molecular determinants of pathogen virulence and host susceptibility to uncover targets for genetic and molecular disease management innovations. Of particular interest have been *Xanthomonas* genomic loci shaped by host-pathogen co-evolution, loci encoding fitness traits, and corresponding host targets. Genetic and molecular research on *Xanthomonas* has spanned nearly 50 years, from early genetic and molecular studies enabled by Sanger sequencing and recombinant DNA technology to recent large-scale comparative and functional genomics of both pathogen and host, empowered by high-throughput sequencing and targeted mutagenesis.

Several excellent review articles have covered this work in detail [4–11]. Our aim in this paper is to synthesize recent progress in two areas we find most interesting and most promising as foundations for disease management: 1) secretion systems and virulence factors in *Xanthomonas*-plant interactions, particularly their contributions to host and tissue specificity, and 2) plant host resistance to *Xanthomonas*. We begin with an overview of the composition and diversity of the genus, with special attention to variation in host and tissue specificity.

## Yellow units: the genus *Xanthomonas*

The genus *Xanthomonas*, a member of the class Gammaproteobacteria, encompasses species that span a broad range of ecological lifestyles from aggressive plant pathogens to commensal or opportunistic bacteria, reflecting substantial evolutionary and functional diversity within the genus [12]. Cells are Gram-negative rods with a single, polar flagellum. Colonies are typically yellowish white to yellow due to production of the pigment xanthomonadin (from the Greek *xanthós* meaning yellow), and mucoid due to production of the extracellular polysaccharide xanthan gum. Species within the genus have been subject to many taxonomic revisions, and further revisions have been proposed (e.g., [13]), but 39 species are currently recognized [14]. While pathovar is assigned based on host and tissue-specificity and is not a recognized taxonomic designation, comparative genomic analyses are bringing clarity to the evolutionary relationships and in some cases calling for reassignment of named pathovars to a different species (e.g., [15]). Phylogenetically, the genus comprises two major clades, a small, early branching clade that contains primarily vascular pathogens of monocots with unique genetic features, including no or non-canonical type III secretion, and a second clade that contains the vast majority of described plant pathogenic *Xanthomonas* species [16,17].

Vascular pathogens in the genus typically gain access to the xylem through hydathodes or wounds. Entry through hydathodes may occur via guttation fluid exuded at night during plant respiration. Bacterial cells on the leaf surface at or near a hydathode may become suspended in this fluid and carried inside in the morning as photosynthesis resumes, transpiration increases, and negative water potential inside the leaf draws the fluid into the xylem. Entry through wounds, caused for example by herbivores, heavy rain, hail, or mechanical damage, may occur directly if bacteria are present at the wound or, if the leaf surface is wet, following migration to the wound. Once in, *Xanthomonas* can invade the xylem systemically. In some cases, spread from the xylem into other tissues has been documented, for example by the black rot pathogen of cabbage, *X. campestris* pv. campestris (Xcc) [18] and the sugarcane leaf scald pathogen, *X. albilineans* (Xa) [19]. Symptoms of vascular infection include vein discoloration, wilt, chlorosis and necrosis along the veins (which may coalesce, resulting in the hallmark “v”-shaped lesions caused by Xcc in cabbage leaves for example), stunting, and death. The pathogen may be disseminated by splashing rain, herbivores, pollinators, irrigation water, or farm equipment. Also, some vascular *Xanthomonas* colonize and can reinitiate infection from the seed; this too is an important mode of dissemination [20,21]. *Xanthomonas* can also overwinter in plant debris. In addition to Xcc, Xa, and the banana wilt pathogen *X. vasicola* pv. musacearum (Xvm) alluded to earlier, examples of vascular pathogens important economically and/or as models include the rice bacterial blight pathogen *X. oryzae* pv. oryzae (Xoo), the cassava bacterial blight pathogen *X. phaseoli* pv. manihotis (Xpm), the cotton bacterial blight pathogen *X. citri* pv. malvacearum (Xcm), the forage grass wilt bacterium *X. translucens* pv. graminis (Xtg), and *X. translucens* pv. translucens (Xtt), which causes bacterial leaf streak, blight, and black chaff of barley.

Non-vascular *Xanthomonas* enter leaves through stomates or fruit through lenticels. They may also gain access to the mesophyll through wounds. These pathogens are characterized by their inability to move systemically in the plant. For example, even when directly inoculated to cut leaf tips, the rice bacterial leaf streak pathogen *X. oryzae* pv. oryzicola (Xoc) is unable to invade the xylem, unlike its close vascular relative Xoo [22]. Under conducive conditions, however, non-vascular pathogens can produce large, spreading lesions and disseminate locally to cause damage on par with vascular pathogens. Lesions often begin as chlorosis then advance to necrosis. Depending on the host leaf anatomy, lesions may develop as spots or (interveinal) streaks. Some non-vascular *Xanthomonas*, like the citrus canker pathogen *X. citri* subsp. *citri* (a monophyletic subspecies no longer designated as pathovar; often abbreviated Xcc but here as Xc-c to distinguish it from the black rot pathogen) and the soybean pustule pathogen *X. axonopodis* pv. glycines (Xag), cause hyperplasia and or hypertrophy at infection sites, resulting in eruptive lesions. Such lesions have been proposed to play a role in pathogen dissemination [23]. Absent such lesions, bacteria may exude back to the leaf surface through stomates from which, like vascular pathogens, they may be disseminated by rain, insects, animals, or machinery. In addition to Xoc, Xc-c, and Xag, non-vascular *Xanthomonas* important economically and or as models include *X. euvesicatoria* (Xe), *X. vesicatoria* (Xv), *X. perforans* (Xp), and *X. hortorum* pv. gardneri (Xhg), each of which causes bacterial spot of tomato and pepper; *X. campestris* pv. raphani (Xcr, formerly pv. armoraciae), a leaf spotter of brassicas; and *X. translucens* pv. undulosa (Xtu), which causes bacterial leaf streak of wheat.

Vascular and non-vascular pathovars of the same *Xanthomonas* species infecting the same or closely related hosts have been the target of comparative studies to understand tissue specificity. These pairs include the rice pathogens Xoo and Xoc [24,25], the brassica pathogens Xcc and Xcr [24,26], and the cereal pathogens Xtt and Xtu [27]. Images of these and other selected diseases caused by *Xanthomonas*, and infection cycles of Xcc and Xoc as representative vascular and non-vascular pathogens, are presented in Figure 1.

**Figure 1.**
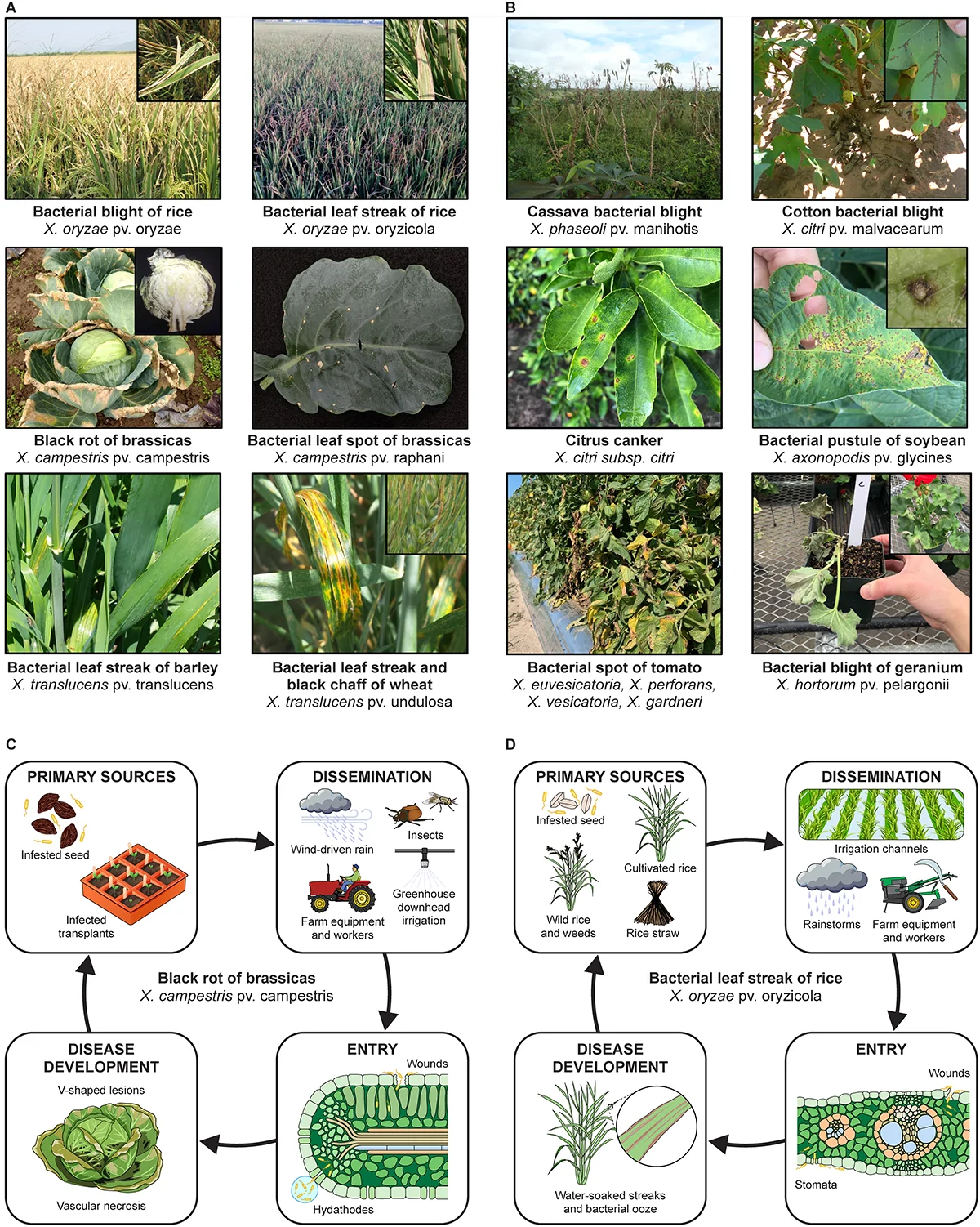
Selected *Xanthomonas* diseases and representative infection cycles. [A] Diseases caused by paired vascular (left) and non-vascular (right) pathovars of three *Xanthomonas* species. [B] Selected diseases caused by other *Xanthomonas* pathogens. [C] Infection cycle of the vascular pathogen Xcc. Xcc can survive in infested soil, seeds, or transplants, in crop debris, and in some weed species. The bacteria are disseminated by wind driven rain, farm equipment, workers, insects, and in greenhouses via overhead irrigation. Once dispersed to host tissue, Xcc enters the xylem through hydathodes via guttation fluid or wounds caused by insects, heavy rain, hail, or mechanical damage. Inside host tissue, Xcc invades the xylem systemically, leading to vein discoloration, wilt, chlorosis and necrosis along the veins that coalesce into “v”-shaped lesions, ultimately leading to stunting, and in severe cases, death. [D] Infection cycle of the non-vascular pathogen Xoc. Xoc can survive in infested seeds, rice straw, wild and cultivated rice, or weed species. The bacteria are primarily disseminated by rain, machinery, workers, and irrigation, entering host mesophyll tissue via stomata or wounds. Upon infection, Xoc proliferates inside leaf mesophyll tissue, causing interveinal watersoaked lesions that can coalesce into long streaks. Under conducive conditions, Xoc can exude back to the leaf surface through stomates for further dissemination. Photo credits: bacterial blight of rice, A. Bogdanove (Cornell University, USA); bacterial leaf streak of rice, Y.Q. He (Guangxi University, PRC); brassicas, Z. Dubrow (Cornell University, USA); wheat and barley, K. Ledman (University of Minnesota, USA); cassava, C. Trujillo (Universidad de los Andes, Colombia); cotton, T. Wheeler (Texas A&M University, USA); citrus, N. Wang (University of Florida, USA); soybean, D. Mueller and A. Robertson (Iowa State University, USA); tomato, N. Potnis (Auburn University, USA); geranium, J. Chow (Ohio State University, USA).

## *Xanthomonas* virulence mechanisms: clues to their host and tissue specificity

### Surviving in the phyllosphere

*Xanthomonas* species initiate infection as epiphytes on the leaf surface, or phyllosphere, a harsh environment characterized by fluctuating nutrient availability, hydration, temperature, and radiation [28]. Although this phase precedes host invasion, epiphytic fitness directly contributes to virulence by determining the population size, persistence, and spatial distribution of bacteria capable of accessing plant entry points. Both vascular and non-vascular pathovars rely on largely conserved mechanisms to persist on the leaf surface, underscoring the epiphytic stage as a key determinant of infection success.

To locate favorable niches on the leaf surface, *Xanthomonas* species rely on motility and chemotaxis, which guide cells toward water films and nutrient rich sites. Nutrient sources like carbon are transiently and heterogeneously distributed in the phyllosphere, posing a major constraint to epiphytic bacterial growth. *Xanthomonas* addresses this constraint through expanded repertoires of high-affinity TonB-dependent receptors and transporters (TBDR/TBDT). These enable uptake of simple sugars such as sucrose and xylose, as well as more complex carbon sources [29,30]. Diversification of TBDR/TBDT repertoires likely reflects adaptation to fluctuating epiphytic nutrient pools and contributes to competitive fitness. Outer membrane vesicles (OMVs) may also play a role in nutrient acquisition to support epiphytic growth. In Xe, OMVs contain proteases, esterases, and xylan-degrading enzymes that may act on substrates on the leaf surface [31]. Finally, mechanisms to protect against osmotic stress driven by wet-dry cycles in the phyllosphere are key. One such mechanism is accumulation by the bacterium of compatible solutes such as trehalose, glycine betaine, and proline, which mitigate osmotic stress [32]. Surface attachment mediated by extracellular polysaccharides (EPS) is central to epiphytic establishment. EPS biosynthesis is encoded by the conserved *gum* gene cluster. EPS promotes hydration, protects cells from desiccation, and stabilizes bacterial aggregates under fluctuating conditions [33]. EPS function is complemented by the type V secretion system (T5SS) adhesins XadA and FhaB, which promote attachment to the host, microcolony formation, and early biofilm development [34]. Biofilm formation enhances desiccation tolerance and provides protection within cuticular microhabitats such as grooves and trichome bases.

In addition to coping with the physical environment on the leaf surface, *Xanthomonas* must compete with other microbes present there. The T4SS mediates contact-dependent interbacterial antagonism, and contributes to competitive fitness [35]. Microbial interactions are also influenced by the T6SS. T6SSs are present across vascular and non-vascular *Xanthomonas* species and also modulate motility, biofilm formation, and virulence [36]. Interestingly, deletion of a core T6SS gene in Xp results in more severe disease, suggesting that the T6SS may act to balance aggressiveness with persistence and dissemination [37]. Comparative analyses reveal that these competitive strategies are evolutionarily plastic, with shifts between T4SS and T6SS-mediated antagonism observed across the genus [38].

### Entering plant tissue

Having established itself on the leaf surface, the next step in infection for *Xanthomonas* is to invade plant tissue. This transition from epiphytic survival to active colonization occurs through wounds caused by mechanical damage, insect feeding, or environmental stress, or through natural openings such as stomata, hydathodes, lenticels, and nectaries. Invasion for active colonization represents a critical bottleneck in the infection cycle.

As introduced earlier, vascular pathovars such as Xcc preferentially exploit hydathodes, specialized leaf-margin organs that directly connect the xylem to the external environment. While essential for plant water homeostasis, hydathodes constitute a vulnerability because these water pores do not fully close in response to abiotic or biotic cues [39], and cycles of water guttation and absorption through them can carry surface bacteria inside leaf tissue. Although structural defenses at hydathodes are lacking, in *Arabidopsis* it has been shown that a strong post-invasive immune response by cells in the epithem at the base of the pore presents a barrier. Defense at this site is mediated primarily through the cell-surface co-receptor BAK1 and the intracellular EDS1-PAD4-ADR1 signaling complex [40]. Factors key to overcoming this barrier have been identified in the hydathode adapted pathogen, Xcc, by systematic transcriptome profiling and barcoded transposon insertion sequencing [41,42]. Early during hydathode colonization, genes associated with motility are downregulated, suggesting a transition to a sedentary state. Within the epithem, T3SS effectors and T2SS-secreted cell-wall degrading enzymes (CWDEs) are deployed to promote bacterial multiplication and suppress immune signaling. During this colonization, transporters for carbohydrate and phosphate uptake and sulfur assimilation pathways are upregulated, ostensibly to support Xcc growth and counter any nutrient limitation imposed by the host as part of the defense response. Following establishment in the epithem, barrier breach to access the xylem represents a critical transition in disease progression. CWDEs, including CbsA, are specifically required for xylem entry, enabling Xcc to spread systemically through the vascular network [41,43].

Non-vascular pathovars, in contrast, primarily exploit stomata as entry points, although successful entry depends on host genotype, environmental conditions, leaf developmental stage, and epiphytic population density. Multiple type III secreted effectors (T3Es) act convergently on stomatal guard cell signaling. XopS from Xe prevents PAMP-triggered stomatal closure in pepper through manipulation of WRKY40 stability and alteration of salicylic acid and jasmonic acid defense hormone crosstalk [44]. XopAP from Xoc binds phosphatidyl inositol 3,5 biphosphate, a membrane phospholipid, and, by competing with vacuolar H^+^-pyrophosphatase for phospholipid binding, inhibits vacuolar acidification in guard cells and prevents stomatal closure [45]. The *X. fragariae* effector XopK interferes with abscisic acid accumulation and signaling, blocking stomatal closure induced by abscisic acid and methyl jasmonate [46]. These functionally convergent strategies illustrate how non-vascular *Xanthomonas* pathogens reliably convert a host-regulated barrier into a permissive entry route.

### Manipulating the plant immune system

Upon accessing the leaf interior, *Xanthomonas* undergoes rapid transcriptional reprogramming to colonize its preferred niche, the mesophyll apoplast for non-vascular pathovars, or the xylem vessels for vascular pathovars [6]. Entry alone, however, does not guarantee successful colonization. Rather, the ability to survive induced host defenses and proliferate post-entry ultimately determine the infection outcome. Inside the host, *Xanthomonas* encounters a multilayered plant immune system conventionally conceptualized as two interconnected layers, described in detail later in this review. Briefly, the first layer, pattern-triggered immunity (PTI) represents a defense response initiated upon recognition of conserved microbe-associated molecular patterns (MAMPs) in the extracellular space by host plasma membrane-localized pattern-recognition receptors (PRRs). In addition to PAMPs, PTI may be triggered by plant cell damage-associated molecular patterns (DAMPs). The second layer, effector-triggered immunity (ETI) is activated when specific pathogen effector proteins injected into plant cells via the bacterial T3SS are recognized by intracellular resistance proteins, resulting in localized plant cell death and restriction of pathogen growth.

A variety of *Xanthomonas* enzymes secreted via the conserved *xps* and *xcs* T2SSs contribute to infection by facilitating tissue penetration and nutrient acquisition. These include several types of CWDEs such as pectinases, xylanases, esterases, and glucanases [47], but also proteases. Activity of these enzymes generates DAMPs. The enzymes thus can promote host invasion but simultaneously increase immune visibility [48]. Consistent with this constraint, comparative genome analyses reveal substantial variation in CWDE repertoires among pathogenic, weakly pathogenic, and commensal *Xanthomonas* strains, indicating that CWDE composition reflects ecological and lifestyle specialization [12][].

Whether PAMP or DAMP activated, PTI represents a major barrier for invading *Xanthomonas*. In many pathogenic strains it is overcome by PTI-suppressing T3Es. Indeed, PTI likely imposed selection for acquisition of the T3SS and the evolution of PTI-suppressing T3Es. Comparative analyses indicate that *Xanthomonas* species cooperatively encode nearly 62 distinct T3E families, reflecting extensive functional specialization and lineage-specific repertoire remodeling across the genus (https://euroxanth.ipn.pt/bacteria:t3e:t3e). T3Es suppress PTI through an array of mechanisms, including direct interference with immune signaling components, perturbation of protein turnover, and manipulation of host cellular processes required for defense activation. Well-characterized PTI suppressors include XopQ, XopX, XopZ, and XopN, which collectively inhibit PTI [48]. Additional effectors such as XopL, an E3 ubiquitin ligase, and XopR attenuate PTI signaling by targeting host regulatory hubs involved in immune activation and signal amplification [49,50]. Other effectors, including XopK, XopP, XopS, XopH, XopAU, XopD, XopJ, also contribute to PTI suppression [4].

Just as MAMPs and type II-secreted enzymes may betray pathogen presence and trigger PTI, some T3Es may become recognized by the host and trigger ETI. Not surprisingly, however, several T3Es have been identified that directly interfere with ETI. Some of these target ETI signaling components. One example is XopQ, which inhibits Bs2-mediated ETI in response to AvrBs2 in pepper and Xv3-mediated ETI in response to AvrXv3 in tomato by targeting components of the ETI-associated MAPK cascade and 14–3-3 isoform TFT4 [51]. Another is AvrBsT, which interacts with SNF1-related kinase 1 (SnRK1), a regulator of sugar metabolism required for efficient induction of AvrBs1-triggered immunity in pepper [47]. Other T3Es interfere with ETI by interacting with the effectors that trigger ETI. An example is XopG, which interferes with XopQ- and XopX-triggered immunity in rice. The plant recognizes XopQ and XopX together in the nucleus, but XopG prevents recognition (and ETI) by sequestering XopQ and XopX in the cytoplasm [52]. XopU, XopV, XopP, and AvrBs2 have also been shown to interfere with XopQ- and XopX-triggered immunity in rice [52]. The mechanisms are not yet understood, but evidence for XopP and AvrBs2 in Arabidopsis and tobacco, respectively, suggests that these effectors are general suppressors of ETI that act on plant components [53,54]. Considering these examples, ETI suppression appears to have evolved redundantly and iteratively, highlighting strong selective pressure to counteract host immune recognition across different hosts.

The extent to which different *Xanthomonas* strains can suppress, evade, or tolerate plant immunity largely determines their pathogenicity. Commensal or opportunistic strains may partially suppress PTI yet fail to overcome ETI, whereas pathogenic strains typically encode large and specialized effector repertoires capable of countering both defense layers. In some cases, failure to bypass ETI directly constrains host range. For example, the effector AvrBsT enhances the fitness of Xp in tomato but triggers resistance in pepper [55]. Similarly, the broadly conserved effector XopQ acts as a host range determinant in *Nicotiana* species, where its recognition contributes to nonhost resistance [56]. Recent work highlights that these pathogenicity-determining interactions are not only genotype-dependent but also spatially constrained at entry sites such as hydathodes. Although hydathodes are gateways into the vascular system, they are actively guarded by the immune hubs indicated earlier. These defenses limit bacterial multiplication within hydathodes and bacterial escape into the xylem. While adapted pathogens can advance into the xylem through action of CWDEs, non-adapted pathogens are more effectively confined and thus unable to do so. A second, hormone-dependent layer involving pipecolic acid and salicylic acid further constrains vascular spread [40]. The conceptual framework illustrated in Figure 2 depicts plant immunity as sequential layers of defense, which not all *Xanthomonas* strains are equipped to traverse.

**Figure 2.**
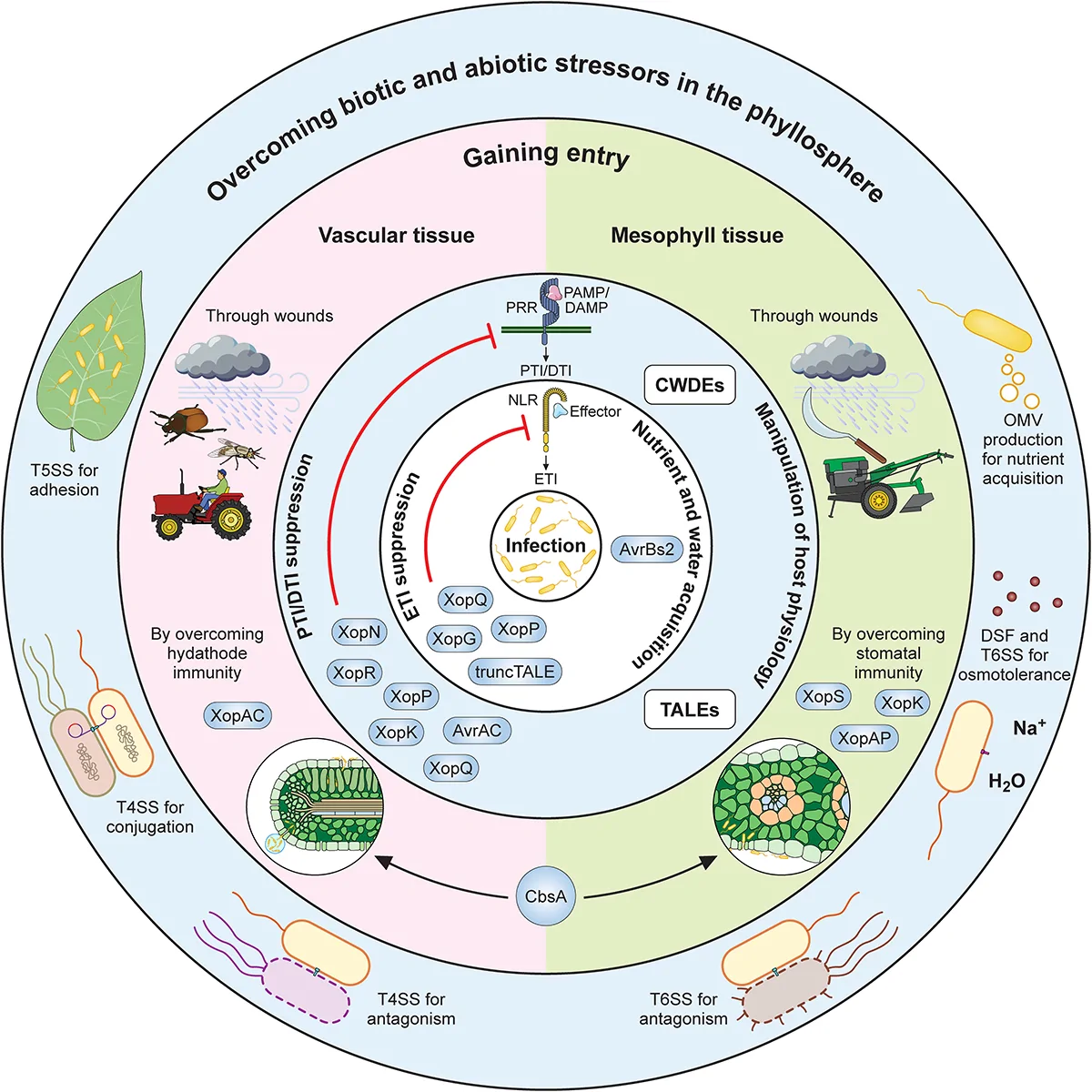
*Xanthomonas* overcomes numerous environmental and host immune barriers to infect plants. Upon landing on the leaf surface, *Xanthomonas* encounters biotic (microbial competition) and abiotic (nutrient limitation, oxidative stress, UV) stresses. to survive in this harsh environment, it deploys the type IV secretion system (T4SS) and the T6SS for intermicrobial competition, T5SS for adhesion, T4SS for conjugative transfer of DNA, TonB-dependent receptors or outer membrane vesicles (OMVs) for nutrient acquisition, and DSF and T6SS to coordinate population-level behaviors such as biofilm formation for osmotolerance. Colonization can then occur through natural openings such as hydathodes and stomata, or through wounds. *Xanthomonas* may also deploy proteins through the T2SS, such as cell wall-degrading enzymes (CWDEs) to obtain nutrients and/or facilitate entry at this stage. The next barrier is pattern-triggered immunity (PTI), a defense that is mediated by plant cell surface pattern-recognition receptors (PRRs) and that can result in generation of reactive oxygen species, cell wall fortification, and production of antimicrobial compounds. PTI also drives stomatal closure, referred to as stomatal immunity. Patterns may include microbial molecules or pathogen-dependent plant-derived molecules. Certain *Xanthomonas* strains evade PTI by having evolved variant “patterns” not recognized by the plant. *Xanthomonas* may also prevent or inhibit PTI through the action of effectors delivered through the T3SS (T3Es). Commensal strains may exhibit limited capacity to overcome PTI and remain restricted to this stage. Effector-triggered immunity (ETI) represents the final barrier. Driven by plant intracellular nucleotide-binding leucine-rich repeat receptors (NLRs) that detect T3Es, ETI shares features and signaling components with PTI, and characteristically culminates in the hypersensitive response (hr), a form of programmed plant cell death. ETI is a major component of hydathode immunity, acting against pathogen entry. *Xanthomonas* may overcome the ETI barrier by having no recognized effector, or by having evolved one or more effectors that, via interaction with components of the ETI pathway or with the recognized effector(s) themselves, block recognition or signaling. Fully pathogenic strains suppress both PTI and ETI. in addition to immune suppression, T3Es may reprogram host physiology to promote watersoaking, liberate nutrients, or otherwise facilitate colonization and/or dissemination. A major class of *Xanthomonas* T3E that do this are the transcription activator-like effectors (TALEs). TALEs reprogram plant physiology by directly upregulating effector-specific plant “susceptibility” genes (S genes). Collectively, T2SS and T3SS effector repertoires are proposed to be the predominant determinants of the ability of *Xanthomonas* strains to traverse host immune barriers, thereby shaping colonization capacity, tissue specificity, and host range.

### Rewiring host physiology

T3Es play a central role in suppressing host immunity. But they also rewire host physiology to promote pathogen proliferation in other ways. XopD is a SUMO protease that acts on host proteins to reshape host transcriptional programs and hormone responses, specifically defense-associated senescence and ethylene responses, prolonging the association with living host tissue and increasing bacterial population growth [57]. Effectors like AvrBs2, which contains a glycerophosphodiesterase domain, can, upon delivery into plant cells, catalyze the conversion of uridine 5’-diphosphate- α-D-galactose into the sugar xanthosan (phosphodiester, bis- [1,6]-cyclic dimeric α-D-galactose-phosphate). Xanthosan is subsequently released into the extracellular space, where it can be catabolized by the bacteria [58]. Transcription activator-like effectors (TALEs), which are host-nuclear localizing, DNA-binding proteins, upregulate transcription of effector-specific “susceptibility” (S) genes, which in turn promote pathogen colonization of the plant, cause symptoms, or both [11]. S genes identified to date include nutrient transporters, plant cell-wall modifying enzymes, and transcription factors, including developmental regulators. The postulated or demonstrated roles of these S genes include supporting pathogen metabolism, modulating host cell wall properties to promote watersoaking and lesion expansion, and facilitating pathogen egress and dispersal through eruptive lesion development [11]. An example in citrus is particularly interesting because it demonstrates synergy between the bacterial T3- and T2SSs in obtaining nutrients from the host. The S gene *CsLOB1*, activated by the Xc-c TALE PthA4 and encoding a transcription factor, in turn activates host genes involved in fruit ripening, including many CWDE genes [54], resulting in release of sugars from the plant cell wall. Among these sugars, xylose, a component of the hemicellulose xylan, activates expression of a T2SS-secreted xylanase in the bacterium. Using this enzyme, Xc-c degrades xylan for uptake and utilization. PthA4 and the other examples presented here illustrate that, while T3Es are commonly perceived as functioning primarily in counter-defense, including against DAMPs generated by bacterial T2SS-secreted enzymes, they contribute to disease in many other ways, including through synergy with T2SS-secreted enzymes.

### Colonizing specific hosts and tissues

Defining host specificity determinants in *Xanthomonas* remains an active area of investigation despite the availability of extensive genomic resources [6]. While an individual T3E can render a strain avirulent by activating ETI in a particular genotype of a particular host species, its loss or mutation may or may not lead to an expansion of host specificity. The effector itself might be required for virulence in the new host species, or a distinct, positive acting host-specific virulence factor might be required. Early studies investigating host range expansion moved the field away from single gene explanations toward a repertoire-for-repertoire hypothesis. Correlations between host range and the T3E repertoires (“effectoromes”) supported this hypothesis, where multiple virulence factors collectively influence the ability of bacteria to colonize specific plant hosts, and in turn, host-driven immune selection acts as a major force shaping effectorome composition [59]. Subsequent studies, however, demonstrated that host specificity cannot fully be explained by T3E content alone, requiring a more holistic view. Comparative genomics and genome-wide association analyses of pepper-infecting *Xanthomonas* strains revealed contributions from additional loci, including TonB-dependent receptors and adjacently encoded CWDE loci [60]. Variation in MAMPs may also contribute to host specificity, shaped by the evolving landscape of PRRs across plant taxa. Likewise, variation in chemotactic sensors, attachment mediators, and transcriptional regulators may act as ecological filters influencing host specificity, with variation in the respective cognate host molecules shaping the evolution of that specificity over evolutionary time [6]. Host specificity in *Xanthomonas* is thus best understood as a multigenic trait. Continued studies incorporating approaches such as functional genomics and experimental evolution will be essential to decipher host specificity determinants.

In addition to host specificity, tissue specificity is an axis of specialization. Tissue specificity reflects adaptations that enable colonization of distinct niches within a plant species, such as vascular or non-vascular tissues. These different tissues present unique structural, chemical and immune environments. Comparative analysis of pathogenesis-associated gene clusters across diverse *Xanthomonas* strains differing in tissue tropism failed to identify gene gain or loss polymorphism associated with tissue specificity. However, positive selection was detected in components of the *xcs* T2SS and the *hrp* T3SS, and at a small number of amino acid positions in the T2SS protein XpsD and the T3SS-associated protein HpaA variation correlated with tissue specificity. Thus, tissue tropism in *Xanthomonas* is likely shaped by fine-scale functional diversification within conserved virulence machinery [61] and or lineage-distinct adaptations. An apparent exception is the T2SS secreted enzyme cellobiosidase CbsA. Across several *Xanthomonas* species and pathovars, presence of CbsA is strongly associated with vascular pathogenicity, and in *X. translucens* it functions as a necessary and sufficient determinant of tissue tropism [25]. Gain of *cbsA* enables typically apoplast-colonizing *X. translucens* strains to invade the vasculature, whereas loss of *cbsA* shifts vascular pathogens toward non-vascular colonization.

### Regulating virulence

Virulence in *Xanthomonas* is governed by a highly interconnected regulatory network that links sensing of environmental, host-derived, and population level cues to appropriate pathogenic responses. The regulatory networks controlling virulence have been reviewed extensively [5]. Here we highlight key modules illustrating how *Xanthomonas* uses quorum sensing, global transcriptional regulators, and small RNAs to integrate information on cell density, redox states, nutrient availability, and host cues for dynamic spatio-temporal deployment of virulence factors.

Quorum sensing mediated by the detection of diffusible signaling factors (DSFs) by two component systems (TCSs) plays a central role in coordinating bacterial population-dependent behaviors. DSFs are fatty acid signal molecules generated by the bacterial RpfF synthase. The TCS RpfC sensor histidine kinase undergoes autophosphorylation upon extracellular DSF detection and in turn the RpfG response regulator is phosphorylated to activate its phosphodiesterase activity and to hydrolyze intracellular levels of cyclic di-GMP (c-di-GMP) [62]. High intracellular levels of c-di-GMP bind to the Clp transcriptional regulator, inhibiting Clp binding to virulence gene promoters and blocking upregulation of virulence genes [63]. Upon DSF perception at high bacterial population levels and RpfG activation, c-di-GMP is degraded, allowing Clp to bind target DNA sites and positively regulate virulence genes [64,65]. In addition to virulence gene upregulation, Clp also activates expression of the motility and T3SS regulatory gene *fhrR* and the zinc uptake and export regulatory gene *zur* [66]. Other TCS, such as RavS and RavR, sense different environmental signals but contribute to c-di-GMP turnover in a similar manner. RavS contains PAS domains implicated in oxygen sensing and is presumed to respond to low oxygen conditions [67]. Through TCSs, quorum and redox sensing is coupled to transcriptional control of virulence genes and secretion systems.

At the core of virulence regulation also is the HrpG-HrpX cascade, which coordinates expression of T3SS, T2SS, and other virulence associated genes. HrpG, an OmpR family response regulator, responds to multiple upstream TCSs and environmental stimuli. Upon activation, HrpG induces expression of HrpX, an AraC-type transcriptional regulator. HrpX directly upregulates components of the T3SS machinery and T3Es by binding plant-inducible promoter (PIP) box motifs [68]. The HrpG regulon also extends to genes involved in metabolism, transport, and cell wall degradation, underscoring its role in virulence. HrpG and HrpX regulation is refined by multiple intersecting regulators [69,70]. The transcriptional regulator Zur and HrpG jointly induce *hrpX* expression, while *hrp* gene expression is negatively regulated by FhrR [66,71]. Comparative transcriptomic analyses indicate that while the HrpG-HrpX core is broadly conserved across the genus, the downstream regulon is highly strain- and pathovar-specific. This regulatory plasticity suggests that the evolution of virulence in *Xanthomonas* frequently proceeds through rewiring of regulatory outputs downstream of conserved hubs rather than gain or loss of virulence factors [72]. HrpG can also negatively regulate *rpfG*, indicating coordinated deployment of energetically costly virulence functions in response to both environmental conditions and cell density [73].

In addition to cell density, virulence regulation in *Xanthomonas* is tightly coupled to carbon availability, particularly host-derived sugars. In Xoo, xylose serves as a key signal for HrpX accumulation and *hrp* gene expression, reflecting adaptation to the rice xylem, which is rich in xylan. Xoo likely acquires xylose via xylan depolymerization by T2SS-secreted xylanases, using the sugar both as a carbon source and as a virulence inducer. Induction of *hrp* genes is sugar-specific and not conserved across *Xanthomonas*. For example, in citrus- and tomato/pepper-infecting *Xanthomonas*, galactose, sucrose, and fructose alone induce *hrp* expression, but not xylose, suggesting host-dependent regulatory specialization [74]. The GamR regulator, known for regulating galactose metabolism, directly activates *hrpG* and *hrpX* in Xoo by binding to a shared upstream region. Notably, galactose metabolism-related gene expression mediated by GamR is galactose-dependent, while GamR-dependent activation of *hrp* regulators is galactose-independent, suggesting that *Xanthomonas* has co-opted metabolic regulators for virulence control. In other pathovars, such as Xc-c, galactose itself functions as an inducer of *hrpX* expression [75], suggesting diversification in how conserved regulators interface with host-specific carbon cues.

Post-transcriptional regulation adds a layer of control in virulence regulation. Small RNAs, acting with the RNA chaperone Hfq and RNA binding protein CsrA, enable rapid and reversible modulation of virulence gene expression. Hfq influences expression of T3SS genes, stress response pathways, and metabolic functions. By stabilizing sRNAs and promoting base-pairing with target mRNAs, Hfq enables rapid modulation of regulator abundance and signal amplification downstream of environmental cue sensing. Loss of Hfq results in pleotropic defects in virulence [76]. One such example involves the Hfq-dependent sRNA sx13. sx13 positively influences *hrp* gene expression by modulating the post-transcriptional control of key regulatory transcripts including *hfq* itself. Through this feedback mechanism, sx13 reinforces the Hrp regulatory network and helps stabilize virulence activation once initiated [77]. Such RNA-based reinforcement likely supports sustained T3SS expression during early host colonization, while retaining the capacity for rapid shutdown when conditions change. CsrA adds an additional layer of post-transcriptional control by directly binding to target mRNAs to regulate translation and mRNA stability. In *Xanthomonas*, CsrA impacts motility, EPS production, metabolism, and secretion system output, positioning it as a critical node linking metabolic status to virulence behavior. By coordinating translational repression or activation across multiple targets, CsrA enables *Xanthomonas* to balance growth, persistence, and pathogenicity in response to nutrient availability and stress [78,79].

Thus, by dynamically integrating population density, environmental, and host-derived cues, through interconnected regulatory networks including transcription factors and small RNAs, *Xanthomonas* species spatially and temporally coordinate distinct virulence programs that enable colonization of specific hosts and tissues. Dissecting the infection process of *Xanthomonas* as a stage-specific and niche-adapted process, encompassing epiphytic fitness, entry site exploitation, immune suppression and niche-specific regulation, provides a framework for identifying host targets and pathogen vulnerabilities that can be leveraged to disrupt infection and design strategies to manage the pathogen.

## Plant defense against *Xanthomonas*: understanding and engineering resistance

### The multiple and interconnected layers of plant immunity

For *Xanthomonas* pathogens to colonize their hosts, they must overcome multiple tiers of plant immunity. As outlined previously, PRRs located at the plant cell surface detect PAMPs and DAMPs, which are derived from conserved elements of phytopathogens or from host-derived breakdown products in the apoplast, respectively [80,81]. Recognition of PAMPs or DAMPs by PRRs leads to PTI, which involves ion fluxes including calcium influx, a reactive oxygen species (ROS) burst, induction of defense gene cascades, and callose deposition that together prevent pathogen proliferation and disease progression [80,82]. To overcome this initial plant immune response, *Xanthomonas* (like other Gram-negative plant pathogens) have evolved T3Es secreted into the plant cell that can subvert PTI or otherwise contribute to a suitable environment for bacterial proliferation, leading to effector-triggered susceptibility. Plants in turn have evolved intracellular nucleotide-binding leucine-rich repeat receptor proteins (NLRs) that detect these effectors or their activity. Activation of NLRs initiates ETI, a defense response that converges on programmed cell death, or the hypersensitive response (HR), which restricts pathogen spread [83–85]. Although originally perceived to be separate pathways, PTI and ETI are mutually dependent on and enhance each other; both are required for a robust immune response [86–88]. In addition to PTI and ETI, defense mechanisms specific to *Xanthomonas* have also been described. In this section, PTI, ETI, and *Xanthomonas*-specific defense are discussed, followed by a consideration of the potential for engineered resistance to *Xanthomonas*. Figure 3 depicts the critical players in the multiple and interconnected layers of plant immunity against *Xanthomonas* pathogens.

**Figure 3.**
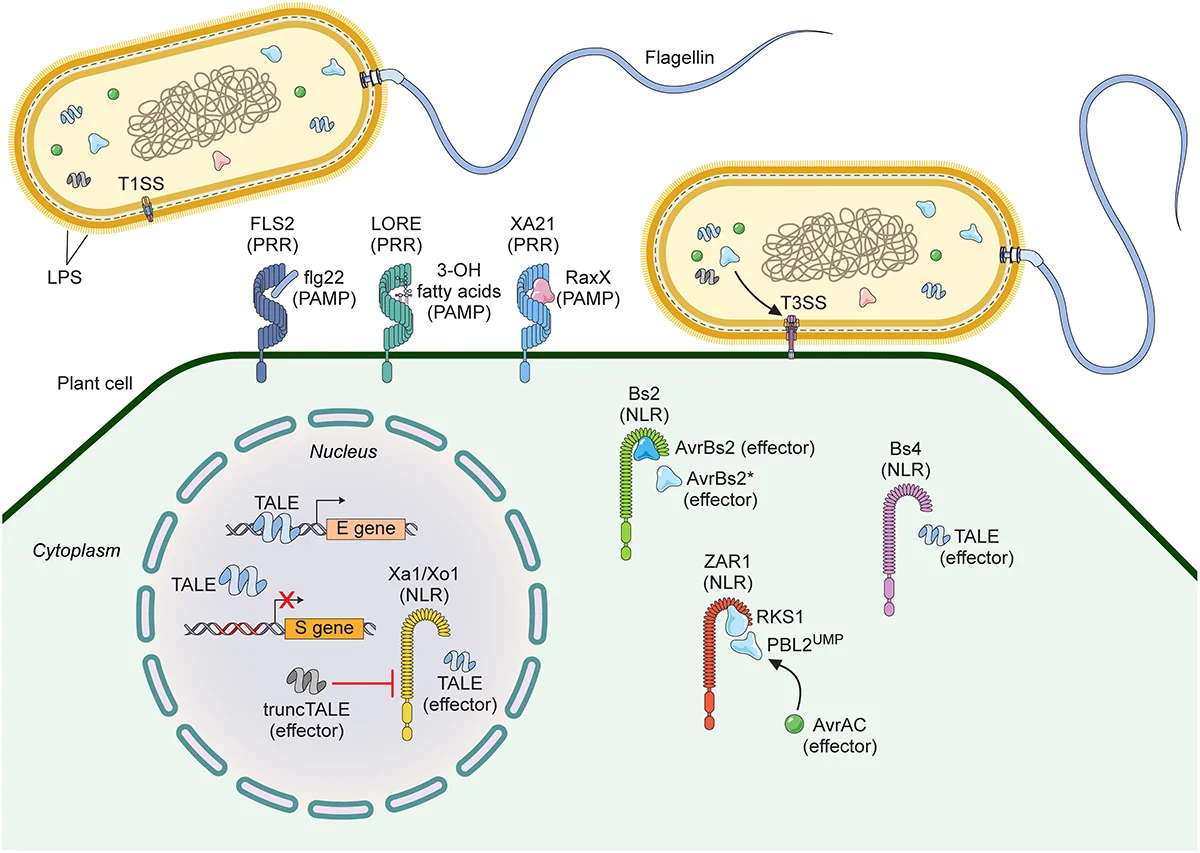
Plant immunity to *Xanthomonas* and its evasion or inhibition by the pathogen. Cell surface PRRs detect PAMPs such as flg22, LPS-associated 3-OH fatty acids, and the secreted peptide RaxX, initiating signaling that leads to PTI. Intracellular NLRs such as Bs2, Bs4, and ZAR1 detect T3Es directly or indirectly, often at the sites of effector action, leading to ETI. *Xanthomonas* may counter ETI through variant T3Es that are not recognized by the cognate NLR, such as AvrBs2* which is not recognized by Bs2. Xanthomonas may also deploy effectors that prevent ETI by interfering with recognition or signaling. TruncTALEs that inhibit TALE-triggered NLRs Xa1 and Xo1 are an example of such T3Es. Two other TALE-specific resistance mechanisms, E genes that block infection by causing localized plant cell death when transcriptionally activated by cognate TALEs, and S gene alleles with promoter mutations that prevent binding and activation by the cognate TALEs, can also confer plant immunity to *Xanthomonas*.

### Defense at the plant cell surface

Cell surface PRRs can be considered the first line of defense that must be overcome by *Xanthomonas* pathogens. The widely distributed FLS2 receptor-like kinase (RLK), for example, detects the flg22 peptide derived from the flagellum which many *Xanthomonas* rely on for motility and plant colonization [89]. The FLS2-1 and FLS2-2 genes of various citrus species detect the Xc-c flg22 and provide a measure of resistance against citrus canker [90]. In Xe, Xp, and Xhg, there is an aspartic acid to valine substitution in the flg22 epitope that abrogates its detection by FLS2 in tomato and pepper. However, the tomato and pepper PRR FLS3 can overcome this loss of detection by detecting a different region of flagellin, flgII-28. In turn, flagella of some species like Xe have an asparagine to lysine substitution in the flgII-28 region that allows escape from FLS3 detection [91]. Another *Xanthomonas*-detecting PRR is the Arabidopsis lipooligosaccharide-specific reduced elicitation (LORE) protein, which specifically detects the lipid A moiety of *X. campestris* lipopolysaccharides (LPS) [92]. The ReMAX protein PRR found in *Arabidopsis thaliana* detects a PAMP called eMax (enigmatic MAMP of Xanthomonas) found in several *Xanthomonas* species [93]. Other conserved *Xanthomonas* molecules, such as the T3SS pilus HrpE protein, the adhesin protein XacFhaB important for host surface attachment and biofilm formation, and peptidoglycan and muropeptides released from *Xanthomonas* cell walls also trigger PTI via yet uncharacterized PRRs [94–96]. In addition to canonical PAMPs derived from conserved bacterial structures, some strain- or species- specific pathogen proteins secreted into the apoplast are detected by PRRs. For example, the rice PRR Xa21 confers resistance to bacterial blight by detecting the RaxX protein secreted by some Xoo strains. RaxX is also found in *Xanthomonas* that infect maize, cassava, and tomatoes [97]. Given the critical roles of CWDEs in infection discussed earlier, it is intuitive that DAMP-activated PTI also plays a role in plant defense against *Xanthomonas*. The Xcc *exbD2* gene, for example, encodes a secreted pectate lyase that releases plant cell wall oligogalacturonides which activate PTI [98]. The diversity of *Xanthomonas* PAMPs and DAMPs and host PRRs, the emergence of PAMPs that escape PTI, and the ability of PRRs to detect species-specific PAMPs point to the critical role these patterns and their corresponding PRR genes play in the disease outcome of the host plant.

### Defense inside the plant cell

*Xanthomonas* pathogens, like other bacterial pathogens, have evolved to obstruct PTI using T3Es secreted into the plant host cell, such as those described in the previous section on virulence mechanisms. However, these proteins can themselves trigger defense through NLR-mediated ETI. Detection by NLRs of well-conserved bacterial T3Es important for virulence can be broadly effective. For example, the pepper NLR Bs2 detects the Xcv T3E AvrBs2, which is required for full virulence in susceptible pepper and alfalfa hosts and is highly conserved across different strains of this pathogen. AvrBs2 is found in other *Xanthomonas* species as well, and Bs2 as a transgene has been shown to protect against Xc-c in citrus and the four *Xanthomonas* species that cause bacterial spot in tomato [99–101]. Another example is the *Nicotiana benthamiana* NLR Roq1. It detects the immune suppressing effector XopQ, which is widely conserved in *Xanthomonas* [102–104].

The detection of effector families containing proteins with shared structures can also confer resistance to multiple bacterial pathovars and, potentially, species. The nuclear rice NLRs Xa1 and Xo1 detect members of the TALE family in Xoc and Xoo [105–108]. Although most Xoc and Xoo strains secrete multiple TALEs with different DNA binding specificities and contributions to virulence, Xa1 and Xo1 are thought to detect diverse TALEs via conserved structural features, since mutated TALEs without transcription activation activity still trigger Xo1 immunity [106]. Interestingly, like the rice NLRs, the tomato NLR Bs4 detects multiple Xe and *X. campestris* pv. amoraciae TALEs. However, in contrast to the rice NLRs which are nuclear, Bs4 is predicted to be cytoplasmic and it is sequence distinct, providing an example of NLRs having evolved convergently to detect an effector family important for *Xanthomonas* virulence [109,110].

In addition to recognizing conserved effectors or effector families with similar structures, some NLRs detect effector activity. The Arabidopsis ZAR1 NLR detects activity of the Xcc AvrAC, which modifies the plant kinase PBL2 [111,112]. ZAR1 is found in a complex with pseudokinases like RKS1. The ZAR1-RKS1 complex detects modified PBL2, which becomes part of the complex, and initiates immunity [112]. Similarly, the ZAR1 ortholog in *Nicotiana benthamiana* detects Xp XopJ4 via XopJ4 modification of the JIM2 pseudokinase [113]. In addition to detecting effector activity, NLRs can be localized or specifically expressed at sites of effector action or pathogen infection. The rice Xa1 and Xo1 NLRs mentioned above are an example. These NLRs reside in the nucleus, where TALEs localize following type III delivery [105,114,115]. The SUT1 NLR provides immunity at the hydathodes where vascular pathogens like Xcc enter the host tissue, contrasting with ZAR1, which is broadly effective against Xcc in the hydathodes, xylem, and mesophyll [116].

How do *Xanthomonas* pathogens overcome ETI? As is the case for PTI suppression highlighted previously, they have evolved both evasion and obstruction mechanisms against ETI. An example of the former is ETI mediated by the pepper Bs2 NLR. Some Xcv field strains have point mutations in the AvrBs2 effector that impair recognition by Bs2 [117]. Bs2 is still the preferred resistance in most tomato cultivars, however, because Xcv strains with Bs2-escaping AvrBs2 effectors show impaired virulence on pepper and tomato hosts and may be less competitive than strains with Bs2-activating AvrBs2 [99]. An example of obstruction can be seen in Xo1 immunity triggered by TALEs. Some *X. oryzae* strains carry truncated TALEs, also called interfering TALEs, that inhibit immunity, potentially by blocking detection of TALEs [107,118].

### Defense mechanisms specific to Xanthomonas

While PRRs guard the apoplast and NLRs the intracellular space, the plant host may also harness effector activity in other ways to mount a defense. TALE activated executor (E) genes illustrate this phenomenon [119]. As noted earlier, in compatible interactions, TALEs bind the promoters of susceptibility (S) genes to induce their expression [11]. E genes are likewise upregulated by TALEs binding to their promoters. Instead of contributing to susceptibility, however, E gene activation results in host cell death and stops the spread of infection, possibly via a type of programmed cell death shared by plant developmental pathways [119]. E genes are found in different plant species. Examples include the *Xa7* gene in rice upregulated by the Xoo TALE AvrXa7 and *Bs3* in pepper upregulated by the Xv TALE AvrBs3 and the Xp TALE AvrHah1 [120–123].

For *Xanthomonas* pathogens that depend on one or more S gene-activating TALEs for virulence, plant defense against them can involve allelic variation at the TALE binding site sequence, preventing S gene activation [11,124]. The genetically recessive rice *xa13* (*OsSWEET11*) and *xa25* (*OsSWEET13*) resistance genes harbor mutations in their promoters, relative to the dominant allele, that prevent TALE binding and gene activation [125–129]. *OsSWEET* genes encode sucrose exporters, and their activation is thought to provide a sugar source for bacteria in the otherwise nutrient poor xylem; lack of *OsSWEET* activation hinders bacterial proliferation and disease [127]. In addition to preventing S gene activation, mutations within the coding sequences of S genes can contribute to defense. One example is the rice *xa5* gene, an allele of the *OsTFIIAγ5* transcription factor subunit gene. TALEs depend on interaction with TFIIAγ5 for full activity. *xa5* encodes a single amino acid substitution relative to the dominant allele that impairs that interaction and reduces the ability of Xoo TALEs to transcriptionally activate *OsSWEET* (and other) S genes, providing resistance through loss of susceptibility [130,131]. Another is the pepper *bs5* gene, encoding a cysteine-rich transmembrane module protein that lacks two amino acids relative to the dominant allele. It confers resistance to Xcv, possibly via disrupted delivery of effectors into the host cell [132].

### Engineered defense

As exemplified above, there are many ways in which *Xanthomonas*, like all pathogens, has evolved and will continue to evolve to overcome plant defenses. Understanding the diverse resistance mechanisms occurring in *Xanthomonas*-plant interactions and how pathogens override them, however, opens the door to molecular interventions to counter pathogen adaptation. One such example centers on TALE-activated S genes. Provided that disruption of the TALE binding site in the S gene promoter has no deleterious effect, S gene editing can be an effective means of engineering resistance to *Xanthomonas* that depend on TALEs [127,133–137]. Due to the modular mechanism of DNA recognition by TALEs, identifying important S genes that could be edited for such loss of susceptibility resistance is relatively straightforward. Candidate targets of a TALE important for virulence can be identified by predicting binding sites across the promoters of all genes in the genome and selecting, among those, genes that are upregulated by the TALE [138]. A new TALE may arise that recognizes the edited site or activates an alternative S gene if present, but complete gene knockout (if not deleterious), or stacking multiple edited promoters, as was done for the *OsSWEET* S gene family, may provide a durable solution [128,139,140].

The battle over nutrients, as exemplified in the TALE and *OsSWEET* interactions, can steer the outcome of infection. Another approach for engineered resistance that targets nutrient availability was demonstrated recently. As noted earlier, the *Xanthomonas* T3E AvrBs2 was discovered to drive in plant cells the production of the sugar phosphodiester xanthosan, which the bacteria use as a carbon source [58]. In rice, transgenic expression of the xanthosan-specific phosphodiesterase XanP effectively counteracted activity of Xoc-delivered AvrBs2, starving the bacterium of this nutrient and conferring resistance to bacterial leaf streak [58].

Yet another way to engineer host resistance is through introduction of an immune receptor from one species into another. Transfer of PRRs such as Xa21 between rice varieties or across species to banana and citrus conferred novel resistance to Xoo, Xvm, and Xc-c, respectively [97,141,142]. The EFR PRR from *Arabidopsis thaliana* to tomato also conferred increased resistance to Xp [143]. And, the maize NLR Rxo1, upon transfer to rice, provided resistance against Xoc [144]. Co-transfer of the pepper Bs2 NLR and its required downstream helper NRCs to rice also conferred resistance to Xoc [145]. Non-NLR genes involved in plant immunity have also been transferred, such as the Hrap and Pflp genes from sweet pepper to banana, in which they conferred robust resistance to Xanthomonas wilt [146]. Together, these examples highlight the utility of mechanistic understanding in one pathosystem for transfer to another.

Host resistance can also be enhanced by knocking out negative regulators. Though de-repression of plant defenses often comes with a growth penalty, in banana, CRISPR mutagenesis of two E3 ubiquitin ligase genes that modulate PTI resulted in plants completely resistant to Xvm with no observed effect on growth [147].

Finally, understanding the molecular mechanisms underlying ligand detection by or inhibition of immune receptors has laid a foundation for recent receptor engineering efforts. An example involves the SCORE PRR from *Citrus maxima* that detects a peptide derived from the bacterial cold shock protein (CSP) csp15. Harnessing diversity among CSPs and corresponding SCORE orthologs that detect them, critical csp15-interacting residues were identified in SCORE. The PRR was then engineered to detect CSPs from various plant pathogens, including multiple *Xanthomonas* species [148]. Although few examples for immune receptor engineering against *Xanthomonas* currently exist, demonstrations of novel PAMP or effector recognition by PRRs or NLRs, respectively, via edited ligand recognition sites, engineered decoy targets for NLRs to catch effectors via their activity, swaps of integrated domains in NLRs, and integration of nanobodies into NLRs, showcase the potential to engineer plant immunity for enhanced resistance [149–155].

## Outlook

As reviewed above, substantial progress has been made in identifying key molecules and mechanisms that determine the outcome of *Xanthomonas* interactions with their hosts. This knowledge has inspired new genetic approaches for mitigating damage caused by the genus worldwide. However, as *Xanthomonas* pathogens continually evolve novel virulence mechanisms to succeed in plants, continued effort to understand those mechanisms, including the virulence factors involved and the plant processes affected, will be critical to guard against the severe losses these bacteria cause. In our view, thwarting pathogen attack by interfering with virulence factor function and by engineering resistance, through both plant breeding and genome editing, are two areas that hold the greatest promise. Essential for these interventions will be a deeper understanding of host and tissue-specificity and the diversity of ways *Xanthomonas* pathogens evade or suppress plant immunity. For the former, elucidation of pathogen and host factors that influence host- and tissue-specificity will continue to be important. Several key questions remain unanswered. How conserved or varied are host- and tissue-specificity determinants in different diseases? To what extent do the nutrient landscapes in different hosts or tissues define infection outcomes? How can some closely related strains differ so dramatically in host range? How do distantly related *Xanthomonas* converge on the same hosts? And, evolutionarily, how plastic are host- and tissue-specificity? For engineering resistance, characterizing T3Es, their targets, and any immune receptors that recognize the effectors will continue to be central. Which effector families or activities are most evolutionarily conserved and therefore the best targets for engineered resistance? How do T3E effector targets function in the normal physiology of the plant and can they be modified to protect against effector activity without perturbing those endogenous functions? What is the mechanistic diversity of effector recognition by immune receptors and what mechanisms are most fine-tunable through protein engineering? Finally, while interaction specificity and immunity merit the greatest focus, there are several other topics that also warrant further attention. An example is how environmental cues are perceived and translated to coordinate virulence factor deployment, e.g. through the T2- and T3SSs. Also, how diversified is that regulation across pathogens of different hosts, and how are responses coordinated at the population level over time and space? Another example, especially important in the context of climate change, is the influence of abiotic factors on *Xanthomonas*-plant interactions, including on virulence, specificity, and immunity. Can we build predictive understanding of that influence to support proactive disease management? Yet another is the effect of the plant microbiome on *Xanthomonas*-plant interactions, and of variation in the microbiome expected with climate change. Can understanding of these effects be exploited for microbiome engineering to control disease? In sum, while a strong foundation has been laid, ongoing research on virulence of *Xanthomonas* pathogens and plant defenses against them represents both a pressing need and an exciting array of opportunities to sustainably improve food and economic security.
